# Identification of immune cells and circulating inflammatory factors associated with neurodevelopmental disorders by bidirectional Mendelian randomization and mediation analysis

**DOI:** 10.1038/s41598-025-98020-0

**Published:** 2025-04-14

**Authors:** Zhiyue Liu, Lihong Wang, Lianhu Yu, Yongheng Zhao, Mengna Zhu, Yu Wang, Aihua Cao

**Affiliations:** Department of Pediatrics, Qilu Hospital of Shandong University, Jinan, China

**Keywords:** Immune cells, Circulating inflammatory factors, Neurodevelopmental disorders, Bidirectional Mendelian randomization, Mediation analysis, Immunology, Diseases, Health care, Medical research, Neurology, Risk factors

## Abstract

The roles of various immune cells and circulating inflammatory factors in neurodevelopmental disorders (NDDs) remain controversial. Therefore we employed a two-sample and bidirectional Mendelian randomization and mediation method to explore the causal relationships between immune cells, circulating inflammatory factors, and NDDs. All data were originated from Genome-Wide Association Study (GWAS) datasets. We found a significant positive causal relationship between 13 immune cells and autism spectrum disorder (ASD), including six CD8+ T cells, one CD3+ T cell, two CD20+ B cells, one CD38+  B cell, and two plasmacytoid DC. 9 inflammatory factors showed significant causal relationships with ASD: interleukins-7 (IL-7), interleukins-2 (IL-2), Interleukin-2 receptor subunit beta levels( IL-2β) and interleukins-18 receptor 1 levels (IL-18-R1) were negatively associated. In contrast, five inflammatory factors were positively associated, such as tumor necrosis factor-α (TNF-α). 14 immune cells exhibited significant causal relationships with attention deficit hyperactivity disorder (ADHD). CD3 on naive CD8br and CD4 on activated Treg were positively associated, while four CD27-expressing B cells were positively associated with ASD. Four CD40-expressing monocytes were negatively associated with ADHD. 7 inflammatory factors had significant causal relationships with ADHD: Fibroblast Growth Factor 23 levels (FGF-23), CD40L receptor levels, Glial Cell Line-Derived Neurotrophic Factor levels (GDNF), TNF-α were more important among these. Mediation analysis identified 12 mediating relationships, with three showing strong evidence: Natural killer cell receptor 2B4 levels (19.9%), FGF-23 (11%), and Eotaxin levels (− 5.95%). Strong causal relationships existed between immune cells, circulating inflammatory factors, and NDDs. Inflammatory factors mediated the pathways between immune cells and NDDs.

**Supplementary Information:**

The online version contains supplementary material available at 10.1038/s41598-025-98020-0.

## Introduction

For the past few years, the global incidence of neurodevelopmental disorders (NDDs) has increased^1^. Epidemiological studies estimate that 1 in 8 children aged 2–9 years is affected by at least one NDD^1,2^, making these disorders a leading cause of childhood disability worldwide. Childhood NDDs include autism spectrum disorder (ASD), attention deficit hyperactivity disorder (ADHD), specific learning disorders, communication disorders, intellectual disability, and motor disorders^3^, which are a range of disabilities due to various disruptions in brain development^4^, with ASD and ADHD representing the most prevalent and clinically impactful subtypes^3^. While ASD is characterized by social communication deficits and restricted/repetitive behaviors^5^, ADHD manifests as inattention, hyperactivity, and impulsivity^6^. Despite their distinct clinical profiles, emerging evidence highlights overlapping genetic and immunological underpinnings between ASD and ADHD, suggesting shared pathobiological pathways^7^.

Currently, the pathogeneses of neurodevelopmental disorders are far from scientifically elucidated^8^. A growing body of evidence implicates systemic immune dysregulation and neuroinflammation as central drivers of NDDs pathogenesis^9^. Recent multi-omics studies and basic research reveal that children with NDD exhibit profound abnormalities in immune cell populations and inflammatory signaling^10,11^. For instance, a 2024 cohort study analyzing 48 immune-related blood biomarkers in 135 NDDs children identified three hallmark features^12^: (1) depletion of the compensatory immunoregulatory system (CIRS), marked by reduced Interleukin-4 (IL-4), Interleukin-10 (IL-10), Soluble interleukin-1 receptor antagonist (sIL-1RA), and Soluble interleukin-2 receptor (sIL-2R); (2) enhanced pro-inflammatory signaling (e.g., elevated TNF-α, IL-6); and (3) imbalanced macrophage polarization (M1/M2 ratio skewing). Also, NDDs are characterized by disturbances in a variety of immune cell pathways, including B cells, T cells, NK cells, and phagocytes. More research on NDDs-related studies was represented by studies on ASD and ADHD, compared to normal people, those with ASD showed various dysregulated immune cells in their blood, cerebrospinal fluid, and brain tissue. These abnormalities included increased B cells, T cells, innate NK cells, and monocytes^13,14^. Further studies also have shown that dysregulated immune cells in ASD, including CD3+  T cells, CD4+  T cells, CD8+ T cells, HLA-DR, CXCR7+ Ki-67+, CD45R+ Ki-67+, CXCR4+ GATA3+, and Th2 lymphocytes^15^. Various immune cells have been shown to contribute to the development of ADHD, including CD8+ cytotoxic T cells, regulatory T cells (Treg cells)^16^, and CD4+ helper T cells^17,18^. Recent basic research has demonstrated that T cells invade the nervous system through the blood-brain barrier, disrupting the functions of microglia and neurons and participating in the pathogenesis of NDDs^12,17^. Secondly, in children with NDDs, autoimmune and allergic diseases were more prevalent, such as asthma and atopic dermatitis^19,20^. Inflammation and immune regulation deficiencies were potential factors in NDDs^21,22^. For example, the levels of inflammatory factors in the plasma of ASD were significantly elevated, such as Interleukin-6 (IL-6), Interleukin-1 beta (IL-1β), Interleukin-12 (IL-12), TNF-α, Interleukin-8 (IL-8), and so on^23,24^. These inflammatory factors could cooperate with immune cells to jointly promote neuroinflammation in children with ASD^25,26^. This process could promote neuroinflammation, alter synapse formation, and affect neuronal function in children with ASD^27^. A recent large-sample analysis revealed a positive correlation between ADHD and circulating inflammatory factors, the most important of which were IL-6, Interleukin-13 (IL-13), Interleukin-16 (IL-16), and TNF-α^28,29^. These inflammatory factors could cooperate with immune cells to jointly promote neuroinflammation in children with NDDs, alter synapse formation, and affect neuronal functions^30^.

While these findings underscore immune dysregulation as a common thread in NDDs, most current studies only focus on specific immune cells and inflammatory factors, without reaching accurate and non-heterogeneous conclusions, nor have they determined the shared immune-related pathways in NDDs^10,11^. Therefore, we intend to conduct a comprehensive analysis of 731 immune cells and 91 circulating inflammatory factors through Mendelian randomization (MR), to explore the causal relationship between immunity and NDDs more systematically and comprehensively and avoid missing important information. Using genetic variation as an instrumental variable, MR mitigates confounding bias and reverse causation, by leveraging the random assignment of genetic variants^31^, thus being more conducive to determining the causal relationship between immunity and NDDs. Additionally, we explored the mediating role of circulating inflammatory factors between immune cells and NDDs.

## Materials and methods

### Study design

**Fig. 1:**
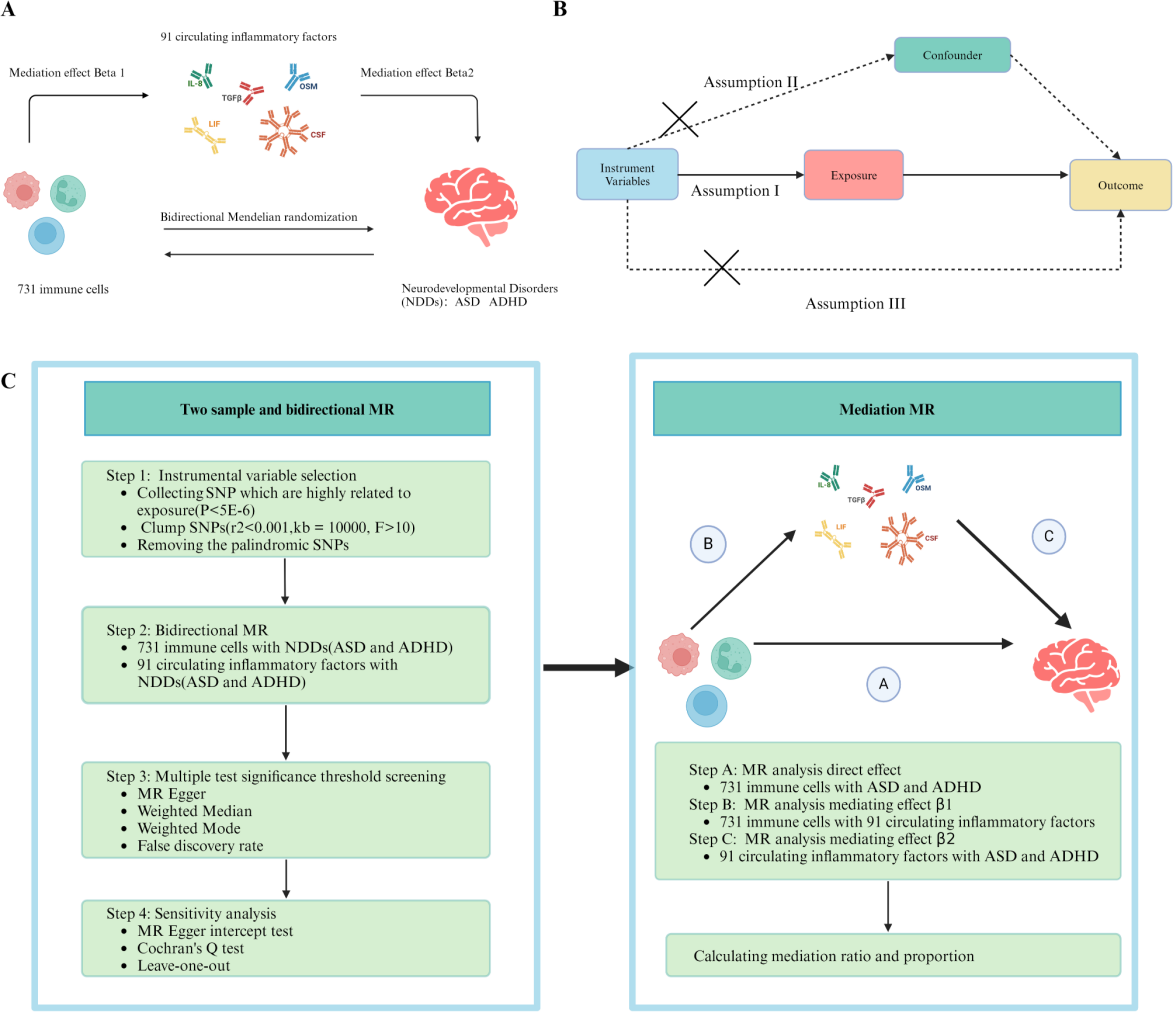
Research design flowchart. (**A**) Overall mediation MR study design, 731 immune cells as exposure, neurodevelopmental disorders (ASD, Autism Spectrum Disorder; ADHD, Attention deficit hyperactivity disorder) as outcome, and 91 circulating inflammatory factors as exposure and mediator. (**B**) Three core hypotheses of MR studies. (**C**) The data analysis process for MR studies.

To scientifically evaluate the causal relationship between 731 immune cells, 91 circulating inflammatory factors, and NDDs (ASD and ADHD). At the same time, by using mediation analysis we evaluated the mediation effect of 91 circulating inflammatory factors between 731 immune cells and NDDs (Fig. 1A). MR leverages genetic variants as instrumental variables (IVs) to infer causal relationships between exposures (immune traits) and outcomes (NDDs). This approach exploits the natural randomization of genetic alleles during meiosis, which mimics a randomized controlled trial in observational data. Genetic variants associated with the exposure are used as proxies to estimate the causal effect of the exposure on the outcome, free from conventional confounding biases and reverse causation. As is shown in Fig. 1B, MR must satisfy three important hypotheses^32,33^: (1) Correlation hypothesis: IVs are strongly related to exposure; (2) Exclusivity hypothesis: IVs can only affect outcomes by influencing exposure, and cannot affect outcomes by other ways; (3) Independence assumption: IVs are not related to confounder.

### Data source

The exposure group data (731 immune cells and 91 inflammatory factors) and outcome data (ASD and ADHD) used in this study employed distinct geographic cohorts and age groups with no participant overlap, satisfying the fundamental requirement for two-sample MR analysis. All clinical participants in the original study used in this study legally signed written informed consent. The specific data sources were as follows: (1) Exposure factors: the raw data of 731 immune cells originated from a 2020 gene sequencing study of 3757 Sardinians aged 20–69 years (Study registration: From ebi-a-GCST90001399 to ebi-a-GCST90002121)^34^. (2) The data for both exposure and mediator factors: the newest 91 circulating inflammatory factors were derived from a 2023 investigation of 14,824 European adults, focusing on genetic loci associated with plasma levels of inflammation-related proteins (Study registration: GCST90274758 to GCST90274848)^35^. (3) Outcome factors: the raw data for ASD were sourced from a 2017 meta-analysis involving 18,381 ASD cases and 27,969 controls exclusively comprising Danish children born 1981–2005, coming from the Psychiatric Genomics Consortium (PGC), diagnosing by ICD-9 or ICD-10 (Open GWAS: ieu-a-1185)^36^. Similarly, the raw data for ADHD came from the PGC study in 2017, encompassing 20,183 ADHD cases and 35,191 controls, exclusively comprising Sweden children born 1987–2006, diagnosed by ICD-9 or ICD-10^37^. All of the above GWAS data went through the following procedures: data quality control, genotype preprocessing, phenotypic preprocessing, association analysis, and result integration. Table 1 provides a comprehensive overview of the specific GWAS datasets employed in the research, detailing their origins and study identifiers.

**Table 1:** Information on GWAS datasets used in the MR study.

GWAS ID	Trait	Data source	Cases	Population	Year	PMID
From ebi-a-GCST90001399 to ebi-a-GCST90002121	731 Immune cells	IEU Open GWAS	3,757	Sardinian adults	2020	PMC8517961
From GCST90274758 to GCST90274848	91 circulating inflammatory factors	Open GWAS database	14,824	European adults	2023	PMC10457199
ieu-a-1185	Autism Spectrum Disorder	iPSYCH-PGC	46,351	Danish children	2017	PMC5441062
ieu-a-1183	Attention Deficit Hyperactivity Disorder	iPSYCH-PGC	55,374	Sweden children	2017	PMC5992329

### Instrumental variable selection

All GWAS summary statistics were obtained from the Open GWAS database (https://gwas.mrcieu.ac.uk/datasets/) and PGC( https://pgc.unc.edu ), which enforces standardized quality control (QC) protocols. We also carried out QC protocol on the downloaded data, and the specific method was as follows. For Single Nucleotide Polymorphism (SNP)-level QC, the database removed variants with high missingness (> 2%), minor allele frequency (MAF) < 1%, and deviations from Hardy–Weinberg equilibrium (HWE; *P* < 1e-10). For sample-level QC, the database excluded individuals with high genotype missingness (> 5%), sex discrepancies (genetic vs. reported sex), heterozygosity outliers (under ± 3 standard deviations), cryptic relatedness (kinship coefficient > 0.125), and population stratification (adjusted via principal component analysis). Detailed QC procedures were described in the Open GWAS documentation and original publications^34–37^. Then, we performed further MR-specific QC, as shown in Fig. 1C. Firstly, a significance threshold of *P* < 5e-6 was used to screen SNPs related to 731 immune cells and 91 circulating inflammatory factors, because SNPs that were selected by a stricter threshold of *P* < 5e−8 were not suited to MR analysis. This method (threshold of *P* < 5e-6) of appropriately relaxing the IVs selection threshold has been widely used in previous studies^38,39^. Additionally, to remove SNPs in linkage disequilibrium, criteria were set at R^2^ < 0.001 and kb = 10,000^40^. Finally, to further ensure the validity and relevance of MR, palindrome SNPs and weak instrumental SNPs (F < 10) were excluded^41^. The F statistic is calculated using the formula: F = R2 × (N − k−1)/k × (1 − R2). Through these three steps, we selected 1,538 SNPs that were closely related to the 731 immune cells (Table S1), and 1,784 SNPs closely related to the 91 circulating inflammatory factors were identified (Table S2). These selected SNPs laid a solid foundation for subsequent MR analysis, enhancing its scientific rigor.

### Statistical analysis

The analysis in this study encompassed two main aspects, as illustrated in Fig. 1C: bidirectional two-sample MR analysis and mediation analysis. It was widely known that Inverse Variance Weighted (IVW) was the primary MR method^42^, other three methods as supplementary. In the lack of pleiotropy, the IVW method provides unbiased and accurate estimates, and an IVW *p*-value < 0.05 is widely recognized for indicating a significant causal relationship^43^. At the same time, we used the False Discovery Rate (FDR) adjustment for the IVW *p*-value, adjusted *p*-value < 0.05 is considered to indicate a significant causal relationship between exposure and outcome^44^. Next, we conducted sensitivity analyses using three methods: leave-one-out analysis, heterogeneity testing (Cochran’s Q test), and pleiotropy testing (MR-Egger intercept test). three methods with a *p*-value < 0.05 indicating the presence of heterogeneity and horizontal pleiotropy, which can affect the stability of the IVW results^45,46^. Pleiotropy was tested using the MR-Egger intercept test, with a *p*-value < 0.05 indicating the presence of horizontal pleiotropy, which can affect the stability of the IVW results^45^. The leave-one-out analysis evaluated the influence of each single SNP on the MR results by removing one SNP at a time^47^.

The main steps of this mediation MR analysis had two steps, shown in Fig. 1C. The mediation effect was calculated as β1 × β2, and the mediation ratio was calculated as (β1 × β2) /direct effect, the mediation ratio represented the proportion of the causal relationship from exposure to outcome that was mediated by the intermediate factors. Based on the mediation effect, we classified the discoverable mediators into different levels of evidence. When only a triangular relationship is present (Fig. 1A), three factors contain a potential mediation role. If there was a triangular relationship and the mediation effect was significantly different from 0, it indicated a significant mediating role. A triangular relationship meant there was a causal relationship between immune cells and NDDs, a relationship between circulating inflammatory factors and NDDs, and a causal relationship between immune cells and circulating inflammatory factors.

All MR analysis progress was made using the R package in R.4.3.2, including “TwoSample MR” and “Mendelian Randomization”.

## Result

### Basic information about instrumental variables and exposures

Following stringent QC and selection criteria (see “Methods” Section), we identified 1538 SNPs robustly associated with 731 immune cell traits (Table S1) and 1784 SNPs linked to 91 circulating inflammatory factors (Table S2) for subsequent MR analyses. The detailed annotations including chromosome location, effect allele, effect allele frequency (EAF), beta coefficients, *p*-values, variance explained, and F-statistics provided in Tables S1 and S2. All SNPs exhibited strong instrument strength, with F-statistics exceeding the recommended threshold of 10. This confirmed that our IVs were unlikely to suffer from weak instrument bias, as F-statistics > 10 ensure minimal attenuation of causal estimates. For transparency, the full distribution of F-statistics was visualized in Table S1 and S2.

### Identification of the causal effect of 731 immune cells on ASD

**Fig. 2:**
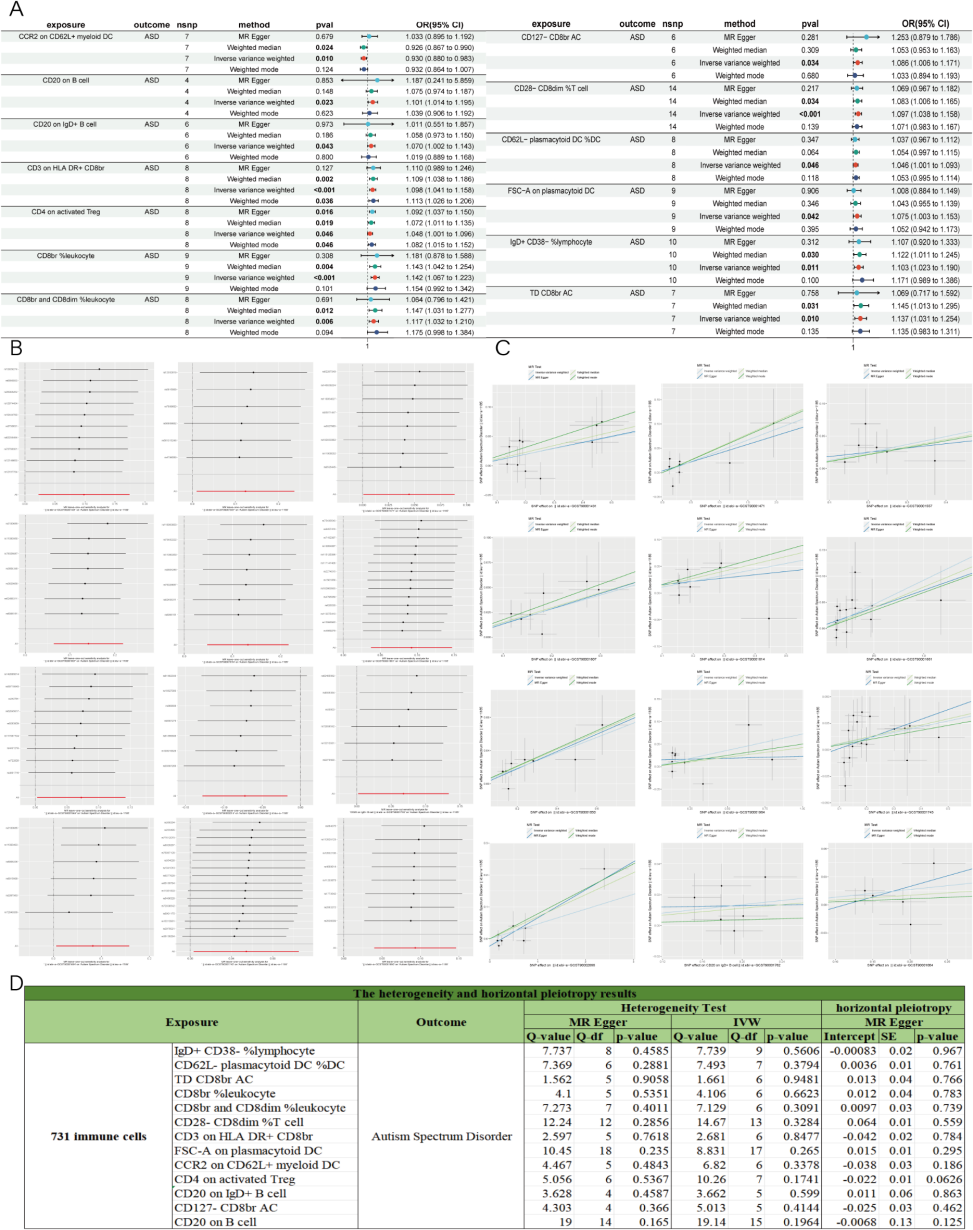
MR results of 731 immune cells with ASD. (**A**) Forest plot of positive MR analysis results between 731 immune cells with ASD. MR, Mendelian randomization; ASD, autism spectrum disorder. (**B**) The results of Leave-one-out analysis between 731 immune cells with ASD. (**C**) Scatter plot of MR analysis results between 731 immune cells with ASD. (**D**) The heterogeneity and horizontal pleiotropy results between 731 immune cells with ASD. Heterogeneity analysis included MR Egger and IVW. Horizontal pleiotropy analysis used the MR Egger intercept method.

We identified 1,538 SNPs associated with immune cells (Table S1). Initial analysis using the IVW method revealed that 13 types of immune cells demonstrated a significant causal relationship with ASD (*P* < 0.05) (Fig. 2A; Table 2). According to the IVW odds ratio (OR) analysis, except for CCR2 on CD62L+ myeloid DC, 12 immune cells showed a positive correlation with ASD risk (OR > 1). The OR directions of these 12 immune cells were consistent across four methods, including IVW, MR-Egger, Weighted Median, and Weighted Mode, indicating their potential role in promoting the incidence of ASD (Fig. 2C). After FDR correction of IVW *p*-values (Table 2), CD4 on activated Treg had an adjusted *p*-value of 0.44002667 (*P* > 0.05), indicating no causal relationship. The remaining 12 immune cells reached statistical significance (*P* < 0.05), suggesting a significant pathogenic association with ASD. Sensitivity and heterogeneity analyses showed no statistical significance (*P* > 0.05) for all results (Fig. 2D). The MR-Egger intercept tests indicated no horizontal pleiotropy (*P* > 0.05), and Cochran’s Q tests suggested no heterogeneity in the MR results (*P* > 0.05). Leave-one-out analysis demonstrated that removing a single SNP did not alter the MR results, with all leave-one-out results presented in Fig. 2B. To validate the causal relationships between the 13 immune cells and ASD, we performed a reverse MR analysis. The IVW analysis of ASD on these 13 immune cells showed no significant reverse causal relationships (*P* > 0.05), as shown in Table S4.

**Table 2:** The main results of all MR analysis.

Exposure	Outcome	Methods	nsnp	*P*	OR (95%)	Adjust *P*
IgD+ CD38- %lymphocyte	ASD	IVW	10	0.011	1.103(1.023–1.190)	0.022
CD62L- plasmacytoid DC %DC	ASD	IVW	8	0.046	1.046(1.001–1.093)	0.048
TD CD8br AC	ASD	IVW	7	0.010	1.137(1.031–1.254)	0.024
CD8br %leukocyte	ASD	IVW	9	0.000	1.142(1.067–1.223)	0.002
CD8br and CD8dim %leukocyte	ASD	IVW	8	0.006	1.117(1.032–1.210)	0.023
CD28- CD8dim %T cell	ASD	IVW	14	0.001	1.097(1.038–1.158)	0.004
CD127- CD8br AC	ASD	IVW	6	0.034	1.086(1.006–1.171)	0.049
CD3 on HLA DR+ CD8br	ASD	IVW	8	0.001	1.098(1.041–1.158)	0.004
FSC-A on plasmacytoid DC	ASD	IVW	9	0.042	1.075(1.003–1.153)	0.041
CCR2 on CD62L+ myeloid DC	ASD	IVW	7	0.010	0.930(0.880–0.983)	0.029
CD4 on activated Treg	ASD	IVW	8	0.046	1.048(1.001–1.096)	0.440
CD20 on IgD+ B cell	ASD	IVW	6	0.043	1.070(1.002–1.143)	0.047
CD20 on B cell	ASD	IVW	4	0.023	1.101(1.014–1.195)	0.037
CD27 on unsw mem	ADHD	IVW	7	0.015	1.084(1.015–1.157)	0.031
CD27 on IgD− CD38dim	ADHD	IVW	4	0.002	1.086(1.031–1.143)	0.024
CD27 on memory B cell	ADHD	IVW	5	0.017	1.088(1.015–1.166)	0.029
HLA DR+ T cell%T cell	ADHD	IVW	4	0.048	0.946(0.895–0.999)	1.000
CD27 on sw mem	ADHD	IVW	7	0.046	1.061(1.001–1.125)	0.050
SSC-A on monocyte	ADHD	IVW	5	0.003	0.949(0.918–0.982)	0.013
CD4 on activated Treg	ADHD	IVW	3	0.010	1.107(1.025–1.196)	0.028
CD40 on CD14+ CD16+ monocyte	ADHD	IVW	4	0.025	0.963(0.932–0.995)	0.035
SSC-A on CD14+ monocyte	ADHD	IVW	4	0.003	0.940(0.903–0.979)	0.019
CD3 on naive CD8br	ADHD	IVW	3	0.033	1.047(1.004–1.091)	0.039
CD40 on monocytes	ADHD	IVW	3	0.014	0.960(0.93–0.992)	0.032
CD40 on CD14- CD16+ monocyte	ADHD	IVW	4	0.021	0.964(0.935–0.995)	0.033
CD40 on CD14+ CD16− monocyte	ADHD	IVW	4	0.010	0.955(0.923–0.989)	0.034
IgD on IgD+ CD24+	ADHD	IVW	4	0.029	0.885(0.792–0.988)	0.037
Fms-related tyrosine kinase 3 ligand levels	ASD	IVW	24	0.023	1.119(1.016–1.233)	0.035
TNF-related apoptosis-inducing ligand levels	ASD	IVW	19	0.022	1.098(1.014–1.19)	0.038
Sulfotransferase 1A1 levels	ASD	IVW	20	0.006	1.109(1.031–1.194)	0.033
Interleukin-7 levels	ASD	IVW	13	0.014	0.858(0.76–0.97)	0.033
Interleukin-2 receptor subunit beta levels	ASD	IVW	9	0.014	0.838(0.727–0.965)	0.030
Natural killer cell receptor 2B4 levels	ASD	IVW	19	0.006	1.144(1.04–1.259)	0.026
Interleukin-2 levels	ASD	IVW	12	0.043	0.874(0.767–0.995)	0.049
interleukin-18 receptor 1 levels	ASD	IVW	19	0.035	1.086(1.006–1.173)	0.048
T-cell surface glycoprotein CD5 levels	ASD	IVW	17	0.016	1.125(1.022–1.239)	0.031
CD40L receptor levels	ADHD	IVW	13	0.004	0.913(0.858–0.972)	0.022
Glial cell line-derived neurotrophic factor levels	ADHD	IVW	7	0.015	1.130(1.024–1.247)	0.034
Eotaxin levels	ADHD	IVW	13	0.024	1.110(1.014–1.216)	0.037
Fibroblast growth factor 23 levels	ADHD	IVW	10	0.016	0.811(0.684–0.961)	0.030
Adenosine Deaminase levels	ADHD	IVW	9	0.047	0.945(0.893–0.999)	0.048
Urokinase-type plasminogen activator levels	ADHD	IVW	16	0.016	0.902(0.83–0.981)	0.031
TNF-related activation-induced cytokine levels	ADHD	IVW	20	0.046	1.080(1.001–1.164)	0.050
FSC-A on plasmacytoid DC	Natural killer cell receptor 2B4 levels	IVW	4	0.021	1.113(1.016–1.218)	0.037
CD27 on memory B cell	Eotaxin levels	IVW	7	0.016	0.953(0.916–0.991)	0.024
CD27 on sw mem	Fibroblast growth factor 23 levels	IVW	10	0.044	0.969(0.940–0.999)	0.048

Overall, all of the above analysis results confirmed a robust and significant causal relationship between 12 immune cells and ASD, including CD62L- plasmacytoid DC %DC, TD CD8br AC, CD8br %leukocyte, CCR2 on CD62L+ myeloid DC, CD8br and CD8dim %leukocyte, CD20 on IgD+ B cell, CD28− CD8dim %T cell, IgD+ CD38− %lymphocyte, CD127− CD8br AC, CD3 on HLA DR+ CD8br, FSC-A on plasmacytoid DC, and CD20 on B cell.

### Identification of the causal effect of 91 circulating inflammatory factors on ASD

**Fig. 3:**
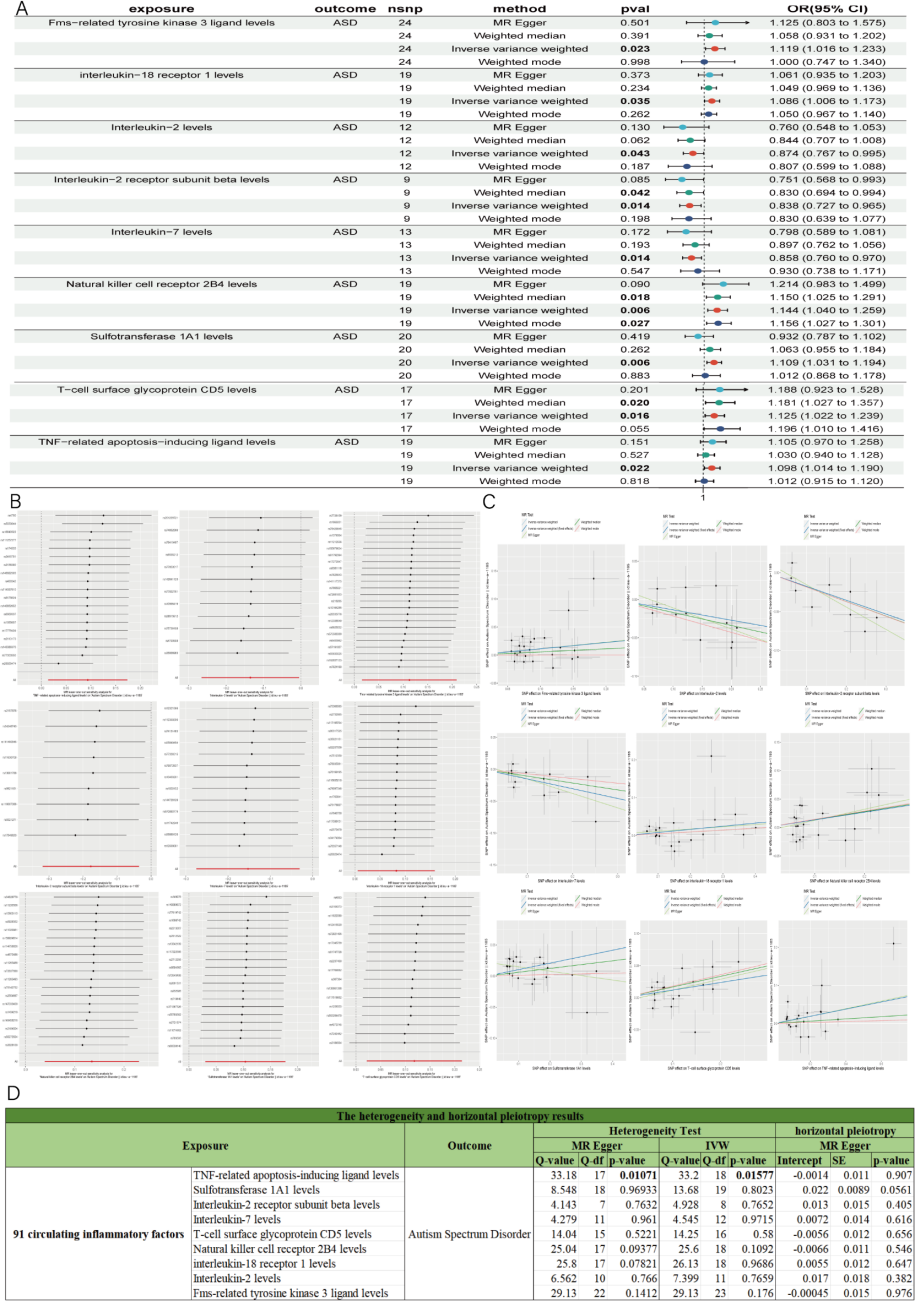
MR results of 91 circulating inflammatory factors with ASD. (**A**) Forest plot of positive MR analysis results between 91 circulating inflammatory factors with ASD. (**B**) The results of Leave-one-out analysis between 91 circulating inflammatory factors with ASD. (**C**) Scatter plot of MR analysis results between 91 circulating inflammatory factors with ASD. (**D**) The heterogeneity and horizontal pleiotropy results between 91 circulating inflammatory factors with ASD. Heterogeneity analysis included MR Egger and IVW. Horizontal pleiotropy analysis used the MR Egger intercept method.

In the second step, we identified 1784 SNPs associated with inflammatory factors (Table S2) and verified the causal relationship between 91 circulating inflammatory factors and ASD. Initial analysis using the IVW method revealed that 9 circulating inflammatory factors demonstrated a significant causal relationship with ASD (*P* < 0.05) (Fig. 3A; Table 2). According to the IVW odds ratio (OR) analysis, 5 circulating inflammatory factors showed a positive correlation with ASD risk (OR > 1), and the OR directions of these 5 factors were consistent across all methods (IVW, MR-Egger, Weighted Median, and Weighted Mode), indicating their potential role in promoting incidence of ASD (Fig. 3C). These factors included Natural killer cell receptor 2B4 levels, Fms-related tyrosine kinase 3 ligand levels, IL-18-R1, T-cell surface glycoprotein CD5 levels, and TNF-related apoptosis-inducing ligand levels. Three circulating inflammatory factors showed a negative correlation with ASD risk (OR < 1), and their OR directions were consistent across all four methods, indicating a protective effect against ASD. These factors included IL-2, IL-2β, and IL-7. Sulfotransferase 1A1 levels showed a positive correlation with ASD, but the MR-Egger result was negative, indicating inconsistency across the four methods. After FDR correction of the IVW *p*-values (Table 2), the *p*-values for the 9 circulating inflammatory factors reached statistical significance (*P* < 0.05), suggesting a significant causal association with ASD. Sensitivity and heterogeneity analyses showed that TNF-related apoptosis-inducing ligand levels had heterogeneity (*P* < 0.05) but no horizontal pleiotropy. Leave-one-out analysis indicated no SNP affecting the robustness of the results (Fig. 3B). For the remaining 8 circulating inflammatory factors, sensitivity and heterogeneity analyses showed no statistical significance (*P* > 0.05) (Fig. 3D). Leave-one-out analysis also indicated that removing a single SNP did not alter the MR results (Fig. 3B). To further validate the causal relationships between the 9 circulating inflammatory factors and ASD, we performed reverse MR analysis. The IVW analysis of ASD on these 9 circulating inflammatory factors showed no significant reverse causal relationships (*P* > 0.05) (Table S6).

Overall, these analyses confirmed a robust and significant causal relationship between 7 circulating inflammatory factors and ASD, including IL-2β, IL-7, T-cell surface glycoprotein CD5 levels, Natural killer cell receptor 2B4 levels, IL-18-R1, IL-2, and Fms-related tyrosine kinase 3 ligand levels.

### Identification of the causal effect of 731 immune cells on ADHD

**Fig. 4:**
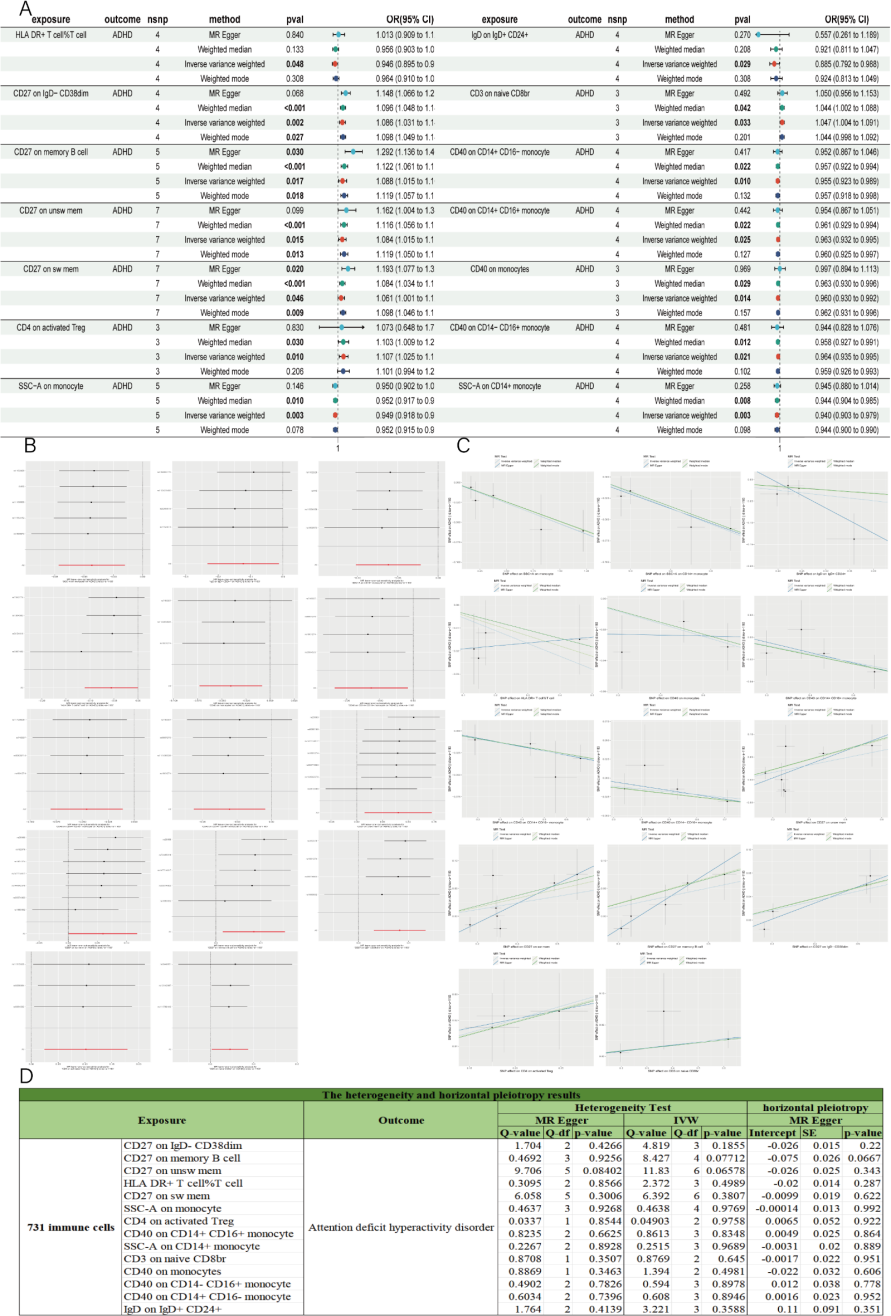
MR results of 731 immune cells with ADHD. (**A**) Forest plot of positive MR analysis results between 731 immune cells with ADHD. MR, Mendelian randomization; ADHD, Attention Deficit Hyperactivity Disorder. (**B**) The results of Leave-one-out analysis between 731 immune cells with ADHD. (**C**) Scatter plot of MR analysis results between 731 immune cells with ADHD. (**D**) The heterogeneity and horizontal pleiotropy results between 731 immune cells with ADHD. Heterogeneity analysis included MR Egger and IVW. Horizontal pleiotropy analysis used MR Egger intercept method.

The IVW results of immune cells and ADHD (Fig. 4A; Table 2) indicated an initial causal relationship between 14 immune cells and ASD (*P* < 0.05). The OR direction of HLA DR+ T cell %T cell was inconsistent across the four analysis methods, and after FDR correction of the IVW *p*-value (Table 2), it did not have a statistically significant causal relationship (*P* > 0.05). The OR directions of the remaining 13 cells were consistent across the four analysis methods, and after FDR correction, the *p*-values for these 13 immune cells reached statistical significance (*P* < 0.05), indicating a significant causal relationship with ADHD. Among these 13 immune cells, 6 immune cells were positively correlated with ADHD risk (OR > 1) (Fig. 4C), including CD27 on IgD- CD38dim, CD27 on sw mem, CD27 on memory B cell, CD4 on activated Treg, CD27 on unsw mem, and CD3 on naive CD8br. 7 immune cells were negatively correlated with ADHD risk (OR < 1), serving as protective factors against ASD, including CD40 on CD14+ CD16+ monocyte, SSC-A on monocyte, IgD on IgD+ CD24+, CD40 on CD14− CD16+ monocyte, CD40 on monocytes, CD40 on CD14+ CD16− monocyte, and SSC-A on CD14+ monocyte. Sensitivity and heterogeneity analyses of the study results (Fig. 4D) showed that the MR-Egger intercept test and Cochran’s test *p*-values for the 14 immune cells were all bigger than 0.05, indicating no heterogeneity or horizontal pleiotropy in the MR results. The leave-one-out analysis also confirmed the robustness of the MR results (Fig. 4B). Subsequently, reverse MR analysis of ADHD and the 14 immune cells showed no reverse causal relationship (*P* > 0.05) (Table S5).

In summary, all these analyses demonstrated a robust and significant causal relationship between 13 immune cells and ADHD, including CD40 on monocytes, CD27 on memory B cell, CD27 on sw mem, CD4 on activated Treg, CD3 on naive CD8br, SSC-A on monocyte, IgD on IgD+ CD24+, CD40 on CD14+ CD16+ monocyte, CD27 on IgD- CD38dim, CD40 on CD14− CD16+ monocyte, CD40 on CD14+ CD16− monocyte, CD27 on unsw mem, and SSC-A on CD14+ monocyte.

### Identification of the causal effect of 91 circulating inflammatory factors on ADHD

**Fig. 5:**
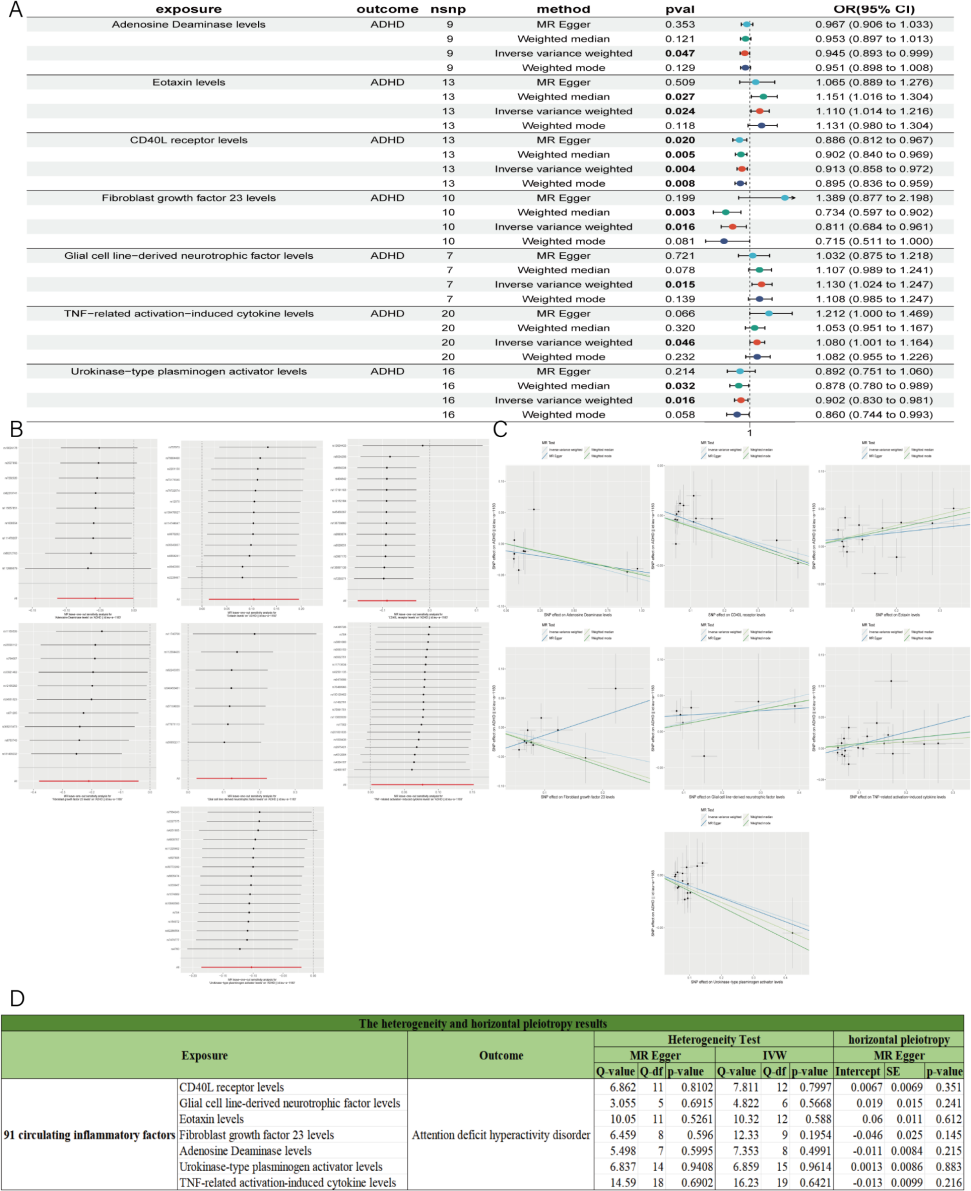
MR results of 91 circulating inflammatory factors with ADHD. (**A**) Forest plot of positive MR analysis results between 91 circulating inflammatory factors with ADHD. (**B**) The results of Leave-one-out analysis between 91 circulating inflammatory factors with ADHD. (**C**) Scatter plot of MR analysis results between 91 circulating inflammatory factors with ADHD. (**D**) The heterogeneity and horizontal pleiotropy results between 91 circulating inflammatory factors with ADHD. Heterogeneity analysis included MR Egger and IVW. Horizontal pleiotropy analysis used the MR Egger intercept method.

The IVW results of circulating inflammatory factors and ADHD (Fig. 5A; Table 2) indicated an initial causal relationship between 7 circulating inflammatory factors and ADHD (*P* < 0.05). After FDR correction, the *p*-values for these 7 circulating inflammatory factors reached statistical significance (*P* < 0.05), indicating a significant causal relationship with ADHD (Table S5 and Table 2). Among these, 3 circulating inflammatory factors were positively correlated with ADHD risk (OR > 1) (Fig. 5C), including Eotaxin levels, GDNF, and TNF-related activation-induced cytokine levels. 4 circulating inflammatory factors were negatively correlated with ADHD risk (OR < 1), serving as protective factors against ADHD, including Adenosine Deaminase levels, FGF-23, CD40L receptor levels, and Urokinase-type plasminogen activator levels. Sensitivity and heterogeneity analyses of the study results (Fig. 5D) showed that the MR-Egger intercept test and Cochran’s test *p*-values for the 7 circulating inflammatory factors were all greater than 0.05, indicating no heterogeneity or horizontal pleiotropy in the MR results. The leave-one-out analysis also confirmed the robustness of the MR results (Fig. 5B). Subsequently, reverse MR analysis of ADHD and the 7 inflammation factors showed no reverse causal relationship (*P* > 0.05) (Table S6).

In summary, all these analyses demonstrated a robust and significant causal relationship between 7 circulating inflammatory factors and ADHD, including Eotaxin levels, GDNF, FGF-23, TNF-related activation-induced cytokine levels, Adenosine Deaminase levels, CD40L receptor levels, and Urokinase-type plasminogen activator levels.

### Identification of the mediation effect of ASD and ADHD

Based on the previous MR results with four different exposures and outcomes, we have confirmed various immune cells and circulating inflammatory factors with strong causal relationships with neurodevelopmental disorders. To determine the mediation effect of circulating inflammatory factors between immune cells and NDDs (ASD and ADHD), we employed the mediation MR method.

**Fig. 6:**
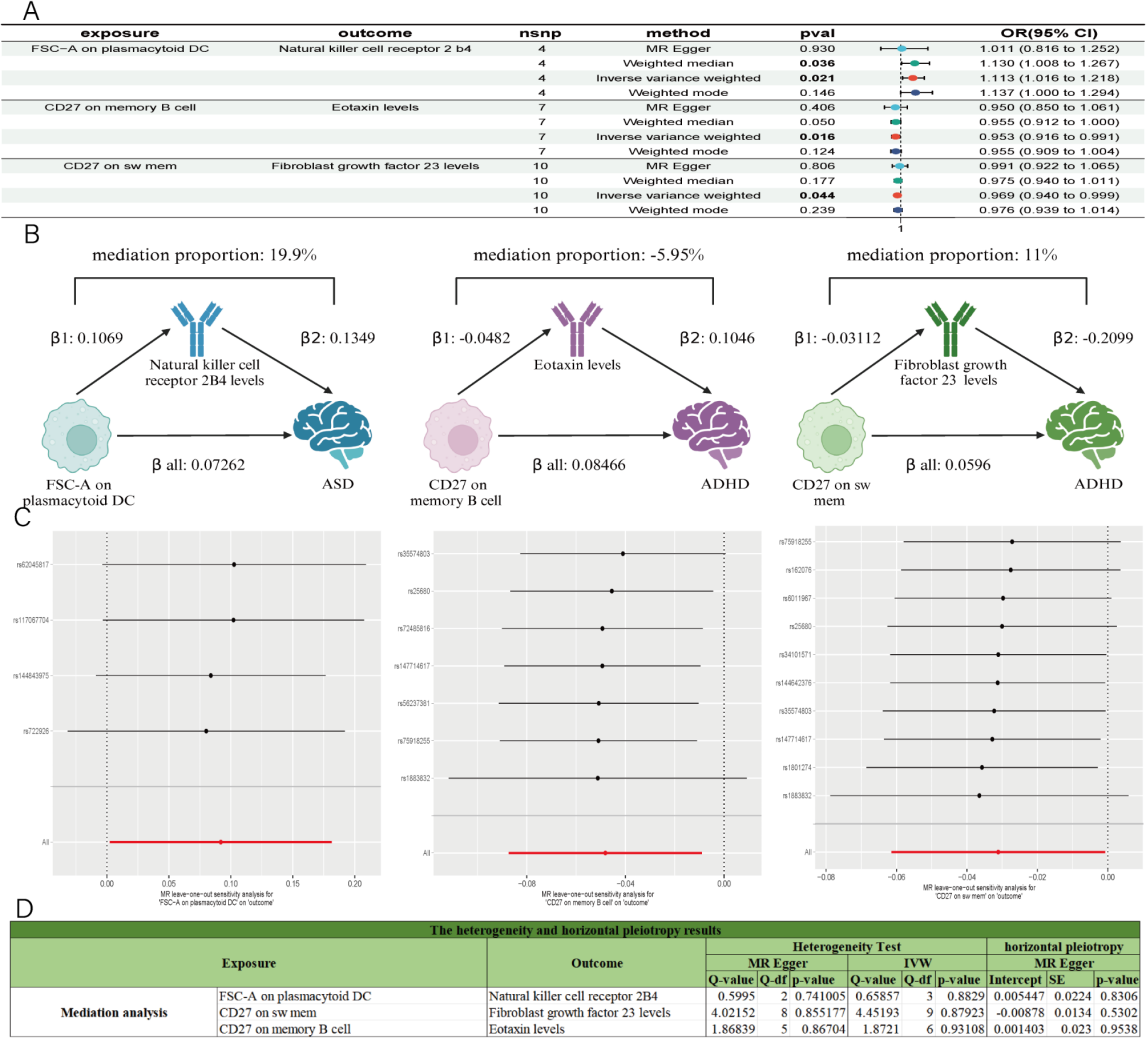
Mediation results. (**A**) Forest plot of positive MR analysis results between 731 immune cells with 91 circulating inflammatory factors. (**B**) The results of mediation MR analysis. (**C**) The results of Leave-one-out analysis between 3 immune cells with 3 circulating inflammatory factors. (**D**) The heterogeneity and horizontal pleiotropy results between 3 immune cells with 3 circulating inflammatory factors.

For ASD, we identified 12 significantly related immune cells and 7 significantly related circulating inflammatory factors. We then selected these 12 immune cells (exposures) and the 7 circulating inflammatory factors (outcomes) for the MR analysis, obtaining the β1 results for immune cells on circulating inflammatory factors. The MR results (Fig. 6A; Table 2) showed that FSC-A on plasmacytoid DC and Natural killer cell receptor 2B4 levels had significant IVW results (*P* < 0.05), and the FDR-adjusted *p*-value < 0.05 (Table S7 and 2). There was no heterogeneity or horizontal pleiotropy (Fig. 6D), and had high robustness of the MR results that confirmed by leave-one-out analysis (Fig. 6C). Therefore, FSC-A on plasmacytoid DC as exposure and Natural killer cell receptor 2B4 levels as mediator with ASD as the outcome formed a triangular relationship. The mediation effect was significant with a mediation proportion of 19.9% (95% CI 1.62%, 41.4%) and *P* = 0.04996888 (Fig. 6B). Additionally, Sulfotransferase 1A1 levels as a mediator between CD28-CD8dim %T cell and ASD also formed a triangular relationship (Table S7) with a potential mediation effect (Table S8), mediation proportion − 14.4% (95% CI − 33%, 4.15%), *P* = 0.127929513.

For ADHD, we identified 13 significantly related immune cells and 7 significantly related circulating inflammatory factors. We selected these 13 immune cells (exposures) and the 7 circulating inflammatory factors (outcomes) for the MR analysis, obtaining the β1 results for immune cells on circulating inflammatory factors. The MR results (Fig. 6A) showed that CD27 on sw mem and Fibroblast growth factor 23 levels had significant IVW results (*P* < 0.05), and the FDR-adjusted *p*-value < 0.05 (Tables S7 and 2). There was no heterogeneity or horizontal pleiotropy (Fig. 6D), and the leave-one-out analysis confirmed the robustness of the MR results (Fig. 6C). Therefore, CD27 on sw mem as exposure and Fibroblast growth factor 23 levels as mediator with ADHD as the outcome form a triangular relationship. The mediation effect was significant with a mediation proportion of 11% (95% CI 0.156%, 21.8%) and *P* = 0.046777517, indicating that Fibroblast growth factor 23 levels significantly mediated the relationship between CD27 on sw mem and ADHD (Fig. 6B). Similarly, Eotaxin levels significantly mediated the relationship between CD27 on memory B cell and ADHD (Fig. 6B), forming a triangular relationship with significant IVW results (*P* < 0.05) and FDR-adjusted *p*-value < 0.05 (Tables S7 and 2). There was no heterogeneity or horizontal pleiotropy (Fig. 6D), and the leave-one-out analysis confirmed the robustness of the MR results (Fig. 6C), mediation proportion − 5.95% (95% CI − 11.3%, − 0.623%), *P* = 0.028573986. Furthermore, we found that CD40L receptor levels potentially mediated multiple immune cells and ADHD (Table S8 and Fig. S1), including, CD27 on memory B cell, CD27 on sw mem, CD40 on CD14+ CD16− Monocyte, CD27 on unsw mem, CD40 on CD14+ CD16+ monocyte, CD27 on IgD- CD38dim, CD40 on CD14− CD16+ monocyte, and CD40 on monocytes.

In conclusion, we identified 12 mediation relationships: 3 strong evidences and 9 potential evidences. Natural killer cell receptor 2B4 levels strongly mediated the relationship between FSC-A on plasmacytoid DC and ASD. Fibroblast growth factor 23 levels significantly mediated the relationship between CD27 on sw mem and ADHD. Eotaxin levels significantly mediated the relationship between CD27 on memory B cells and ADHD. Sulfotransferase 1A1 levels potentially mediated the relationship between CD28-CD8dim %T cell and ASD. CD40L receptor levels potentially mediated the relationships between multiple immune cells and ADHD.

## Discussion

In this study, using MR analysis and GWAS data, we conducted a comprehensive analysis of 731 immune cell types and 91 circulating inflammatory factors in NDDs. As a result, we demonstrated the strong and potential causal relationships between immune cells, circulating inflammatory factors and NDDs (ASD and ADHD), and the mediation role of inflammatory factors between immune cells and NDDs. Immune cells and inflammatory factors play an important role in the pathogenesis of NDDs. This research not only clarified the molecular commonalities and differences related to immunity in NDDs, explored the mechanism of immunity in the pathogenesis of NDDs, but also provided a theoretical basis for precision medicine targeting immune typing.

ASD may be related to immune balance disorder, involving the imbalance of inflammatory factors and autoimmune disorders^9^. In the MR analysis of 731 immune cells and ASD, six T cells were found to be positively correlated with ASD, which were all marked by CD8br, including CD8br %leukocyte, TD CD8br AC, CD8br and CD8dim %leukocyte, CD28- CD8dim %T cell, CD127- CD8br AC, and CD3 on HLA DR+ CD8br. This suggested that CD8+ T cells may greatly promote the onset of ASD. A clinical trial by Lopez-Cacho JM et al. confirmed that the number of CD8+ T cells in ASD patients was higher than in healthy individuals, indicating a positive correlation between ASD and CD8+ T cells^48^. A recent study used BTBR T+ Itpr3tf/J (BTBR) mice to build an ASD model, and the results showed that both peripheral blood and thymus cells of the mice showed higher CD8+ T cells. The pathogenesis of ASD may be the existence of CD8+ T cell-associated autoantibodies, such as dihydrolipoyllysine-residue succinyltransferase component of 2-oxoglutarate dehydrogenase complex, mitochondrial (DLST) and alpha-enolase (ENO1), bound to microglia and drove neuroinflammation^49^. The other mouse experiment demonstrated that CD8+ T cells could lead to ASD by affecting neural progenitor cells, resulting in brain NDDs and ASD behaviors in mice^50^. Another study on postmortem brain tissue of ASD patients provided more direct evidence that immune cells damaged the cerebrospinal fluid (CSF)–brain barrier. Among immune cells, CD3+ and CD8+ T cells were the most prevalent, with a few CD4+ T cells and CD20+ B cells^51^. In brain glial cells, CD8+ T cells produced cytotoxic effector molecules such as granzyme B, causing abnormal membrane vesicles in GFAP+ astrocytes in the brains of ASD patients^51^. ASD children also had increased numbers of CD3+ TIM-3+ and CD8+3 TIM-3+ cells compared to typically developing (TD) controls^52^. Therefore, T cells expressing CD8 and CD3 might be pathogenic immune cells in ASD.

Next, this study found that two types of plasmacytoid DCs were positively correlated with ASD: FSC-A on plasmacytoid DC and CD62L- plasmacytoid DC %DC. A Spain study indicated a significant increase in the frequency of bone marrow DCs in ASD children. Plasmacytoid dendritic cells (pDCs) were associated with the amygdala volume and developmental regression in ASD children^53^. pDCs performed antigen presentation and stimulated other immune cells, primarily through the secretion of inflammatory factors such as IFN-I. Emerging evidence indicates that activation of immune cells such as pDCs triggered the elevation of reactive oxygen species (ROS) and subsequent oxidative stress, a pathological process implicated in ASD-associated neuroinflammation. This mechanism represents a novel pathway in the pathogenesis of neuroinflammatory responses characteristic of ASD^54^. Our results also showed that 19.9% of the causal effect of FSC-A on plasmacytoid DC on ASD was mediated by Natural killer cell receptor 2B4 levels. Therefore, the effect of pDCs on ASD may be partially mediated by inflammatory factors. Then three B cells were positively correlated with ASD: IgD+ CD38− %lymphocyte, CD20 on IgD+ B cell, and CD20 on B cell. CD20 B cells damaged the CSF-brain barrier in ASD brain tissue, but there was no significant difference in B cell counts in peripheral blood^55^, indicating the need for larger clinical studies and basic research to fully confirm the relationship between B cells and ASD.

In the MR analysis of 91 circulating inflammatory factors and ASD, three interleukins—IL-7, IL-2, and IL-2 Rb—were significantly negatively associated with ASD risk, while IL-18R1 and TNF-related apoptosis-inducing ligand (TRAIL) were positively associated with ASD development. Vojdani et al. showed that peripheral blood levels and mRNA expression of IL-2 are lower in children with ASD^56^. Also, IL-2 and IL-7 levels were negatively correlated with stereotypic behaviors^57^ and intellectual scores in ASD^58^. The levels of IL-18 were elevated in the brains of children with ASD, which activated astrocytes and led to neuroinflammation and subsequent cognitive dysfunction^59^. A recent basic study revealed that IL-18 could activate NF-κB to stimulate Fas and Fas ligand (Fas-L) promoter activity while modulating neuronal excitability. These combined effects induce suppression of long-term potentiation (LTP), a hallmark of synaptic plasticity, thereby contributing to learning and memory deficits in ASD^60^. Zhao H et al. reported elevated levels of TNF-α in the peripheral blood of ASD patients^24^ and positively correlated with the severity of ASD symptoms^61^. A co-culture study of mice and human neurons revealed that TNF-α can upregulate glutamate levels, inducing neurotoxicity and promoting neuronal death and apoptosis. Using glutaminase inhibitors can alleviate this neurotoxicity^62^, suggesting potential therapeutic approaches for ASD children.

In the MR analysis of 731 immune cells and ADHD, our results indicated that B cells, monocytes, and T cells had a causal relationship with the onset of ADHD, and these findings were consistent with the results for ASD, suggesting CD8+ and CD3+ T cells were potential common immune cells in NDDs. First, regarding T cells, CD3 on naive CD8br and CD4 on activated Treg were positively correlated with ADHD. Looman KIM et al. found peripheral blood immune cells in 756 children and found that higher levels of helper T cell 1 (Th1) and CD8+ T cells were associated with higher attention problem scores^63^. A turkey case-control study showed a positive correlation between CD3+ CD4+ CD25+ Foxp3+ (Tregs) and ADHD^16^, consistent with our results. A mouse study demonstrated that NCX3-deficient mice exhibit ADHD-like behaviors, attributable to impaired dopaminergic neurotransmission in the prefrontal cortex (PFC). Intriguingly, CD3+ and CD8+ T cells may contribute to this pathogenic mechanism^30^. More basic trials were needed to explore the potential mechanisms between immune cells and ADHD described in this study.

Secondly, our study found that four B cells expressing CD27 were positively correlated with ADHD: CD27 on unswitched memory, CD27 on switched memory, CD27 on IgD- CD38dim, and CD27 on memory B cells. A Stanford University cohort study indicated that children with certain atopic diseases had higher numbers of memory Treg cells, total B cells, and CD27+ IgA+ memory B cells^64^. And these diseases had a strong correlation with ADHD, such as atopic dermatitis^65^ and food allergies^66^. Therefore, ADHD may be closely related to CD27+ B cells. Moreover, subsequent mediation analysis showed that the causal effect of two CD27+ B cells on ADHD was realized through circulating inflammation factors, but further studies were needed in the future.

Thirdly, our results indicated that four monocyte types expressing CD40 were negatively correlated with ADHD, and the inflammatory cytokine CD40L receptor levels were also negatively correlated with ADHD. Avcil S et al. put forward that the monocyte/lymphocyte ratio (MLR) potentially served as a peripheral blood inflammatory marker for ADHD^67^. 1α, 25-dihydroxyvitamin D3 (1,25(OH)2D3) interfered with the effects of CD40L on immunomodulatory and inflammatory responses, so drugs that reduced the amount of 1,25(OH)2D3 may help treat ADHD^68^. Therefore our research suggested that monocytes expressing CD40 and CD40L receptor levels were closely associated with ADHD, and influenced ADHD risk by interacting with multiple inflammatory factors.

Numerous studies have shown that inflammatory factors play a significant role in the development of ADHD, and our results also suggested that these factors could be crucial in ADHD pathogenesis. Firstly, TNF-α was shown to increase the risk of both ASD and ADHD in MR analyses, with clinical data supporting higher peripheral blood TNF-α levels in ADHD patients, correlating positively with hyperactivity symptoms^69^. Moreover, a study utilizing spontaneously hypertensive rats (SHR) as an ADHD model revealed elevated TNF-α levels in the prefrontal cortex (PFC), striatum, and hippocampus. This pro-inflammatory cytokine was found to modulate glutamatergic synaptic strength, thereby impairing cognitive and executive functions, which collectively manifest as ADHD-like behavioral phenotypes^70^. Thus, TNF-α could be a potential pathogenic inflammatory factor in NDDs.

In our MR results, Fibroblast Growth Factor 23 (FGF-23) was protective against ADHD, whereas Glial cell line-derived neurotrophic factor levels (GDNF) promoted ADHD development. Both FGF-23 and GDNF were growth factors that regulated neurogenesis, differentiation, development, gliogenesis, and synaptogenesis, thereby influencing cognition^71^. Bilgic A and Yurteri N all proved elevated GDNF levels and decreased FGF levels in children with ADHD, consistent with our findings^72,73^. In mouse experiments, disruption of the FGFR gene led to increased spontaneous movement and a reduction in cortical inhibitory neurons. FGFR agonists significantly reduced hyperactivity^74^, and pathway analysis confirmed FGFR’s role in ADHD etiology by activating FGFR1b and FGFR2b pathways^75^. Therefore, FGFR agonists and GDNF inhibitors may serve as new therapeutic targets for alleviating ADHD symptoms.

Although the MR analysis in this study provided new insights into the causal relationships between immune cells, inflammatory factors, and NDDs, and offered advantages in reducing confounding and reverse causation, several limitations must be acknowledged. Firstly, all GWAS data used in this study were derived from European populations, which limits the generalizability of our results to non-European populations, particularly given known ethnic disparities in immune profiles and NDD prevalence. Future replication in diverse cohorts is critical to confirm these associations across ancestries. Secondly, this study only established causal relationships between exposures and outcomes, leaving the underlying mechanisms unexplored and necessitating further research. Thirdly, while our sensitivity analyses did not show evidence of pleiotropy or heterogeneity, there may still be unknown confounding factors that could introduce bias into the results. Lastly, although related studies suggest potential interaction pathways for the mediation analysis results, the lack of conclusive literature support requires further validation through clinical trials. Therefore, we plan to make improvements in the future: firstly, we should continuously enrich the GWAS database; Secondly, clinical research and basic research should be continued to further explore causation and mechanism analysis.

## Conclusion

In summary, this study identified causal relationships between immune cells, circulating inflammatory proteins, and NDDs, establishing three mediators with strong evidence. These relationships could serve as valuable biomarkers and potential targets for understanding the biological mechanisms of NDDs and developing new therapies.

## Electronic supplementary material

Below is the link to the electronic supplementary material.

Supplementary Material 1

Supplementary Material 2

Supplementary Material 3

Supplementary Material 4

Supplementary Material 5

Supplementary Material 6

Supplementary Material 7

Supplementary Material 8

## Data Availability

All original data supporting the findings of this study are included in the article and its Supplementary Material. For additional information or inquiries, please contact the corresponding author.
